# Varied Contribution of Phospholipid Shedding From Membrane to Daptomycin Tolerance in *Staphylococcus aureus*

**DOI:** 10.3389/fmolb.2021.679949

**Published:** 2021-06-11

**Authors:** Tianwei Shen, Kelly M. Hines, Nathaniel K. Ashford, Brian J. Werth, Libin Xu

**Affiliations:** ^1^ Department of Medicinal Chemistry, School of Pharmacy, University of Washington, Seattle, WA, United States; ^2^ Department of Pharmacy, School of Pharmacy, University of Washington, Seattle, WA, United States

**Keywords:** lipidomics, Agr, pharmacokinetic and pharmacodynamic model, daptomycin tolerance, phospholipid release

## Abstract

It has been suggested that daptomycin can be inactivated by lipids released by *Staphylococcus aureus* and that this effect is antagonized by phenol soluble modulins (PSMs), which bind to the shed lipids. PSM production is regulated by the Agr system, and others have shown that loss of the Agr function enhances *S. aureus* survival in the presence of daptomycin. Here we assessed the impact of Agr function on daptomycin activity and lipid metabolism under various conditions. Daptomycin activity was evaluated against three sets of isogenic strain series with wild-type or dysfunctional Agr using static daptomycin time-kills over 24 h and against one strain pair using *in vitro* pharmacokinetic/pharmacodynamic (PK/PD) models simulating clinical daptomycin exposure for 48 h. We performed comprehensive lipidomics on bacterial membranes and the spent media to correlate lipid shedding with survival. In static time-kill experiments, two *agr*-deficient strains (SH1000- and USA300 LAC Δ*agrA*) showed improved survival for 8 h compared with their corresponding wild-type strains as seen in previous studies, but this difference did not persist for 24 h. However, four other *agr*-deficient strains (SH1001 and JE2 *agr* KOs) did not demonstrate improved survival compared to isogenic wild-type strains at any time in the time-kills. Lipidomics analysis of SH1000, SH1001, and SH1000- strains showed daptomycin exposure increased lipid shedding compared to growth controls in all strains with phosphatidylglycerols (PGs), lysylPGs and cardiolipins predominating. In the cell pellets, PGs and lysylPGs decreased but cardiolipins were unchanged with daptomycin exposure. The shed lipid profiles in SH1001 and SH1000- were similar, suggesting that the inability to resist daptomycin by SH1001 was not because of differences in lipid shedding. In the PK/PD model, the *agr* mutant SH1000- strain did not show improved survival relative to SH1000 either. In conclusion, inactivation of daptomycin by shed lipids may be dependent on genetic background, the specific *agr* mutations, or the techniques used to generate these KOs rather than the overall function of the Agr system, and its contribution to daptomycin tolerance seems to be varied, transient, and growth-condition dependent.

## Introduction

Daptomycin is a lipopeptide antimicrobial that consists of a cyclic polypeptide with 13 amino acids and a decanoyl fatty acyl tail that plays an important role in the management of invasive infections caused by methicillin-resistant *Staphylococcus aureus* (MRSA). Its mechanism of action involves direct interaction with the negatively charged membrane lipids, phosphatidylglycerols (PGs), leading to loss of membrane potential and cell death (Muraih et al., 2011; Muraih et al., 2012; Pogliano et al., 2012; Bayer et al., 2013). As such, most studies on daptomycin resistance point to development of mutations in genes that control membrane lipid metabolism and/or lead to changes in surface charge, membrane fluidity, or both, such as *mprF*, *cls*, *pgsA*, and the *dlt* operon, which reduces binding of daptomycin to the cell membrane or prevents disruption of the membrane by daptomycin (Yang et al., 2009; Peleg et al., 2012; Mishra and Bayer, 2013; Mishra et al., 2013; Bayer et al., 2014; Cafiso et al., 2014; Mishra et al., 2014; Bayer et al., 2015; Hines et al., 2017; Jiang et al., 2019). Mutations in two-component regulatory systems that regulate cell wall and cell membrane metabolism, such as *vraSR* and *walKR* (Friedman et al., 2006; Mehta et al., 2012; Werth et al., 2021), have also been shown to contribute to daptomycin resistance. We previously applied a novel multi-dimensional lipidomic method to characterizing the detailed lipid profile changes associated with MRSA strains that have developed resistance to daptomycin and found overall greatly decreased levels of PGs in a resistant strain with mutations in both *pgsA* and *mprF* (Hines et al., 2017) and greatly elevated levels of lysyl-phosphatidylglycerols (lysylPGs) and cardiolipins (CLs) in a strain with only an *mprF* mutation (Hines et al., 2020). Thus, altering lipid metabolism is an important route for bacteria to acquire resistance to daptomycin.

In recent years, inactivation of daptomycin by phospholipids released by *S. aureus* upon daptomycin exposure was proposed as a novel mechanism of daptomycin tolerance (Pader et al., 2016). The authors found that this effect may be antagonized by the production of amphipathic peptides called phenol soluble modulins (PSMs), which bind released phospholipids and thus prevent inactivation of daptomycin (Otto, 2014; Pader et al., 2016). PSM production is regulated by the accessory gene regulator (Agr) system, which is encoded by a four-gene operon (*agrBDCA*) and a regulatory RNA gene (RNAIII) (Peschel and Otto, 2013). Pader et al. suggested that the loss of the Agr quorum-sensing system enhances *S. aureus* survival during daptomycin exposure since PSMs are not released, and thus there is no competition for daptomycin sequestration by the shed lipids. However, the detailed composition of membrane lipids and shed lipids by *S. aureus* strains with variable Agr activity in response to daptomycin exposure has not been elucidated and the effect of lipid shedding on daptomycin activity has not been examined under clinically relevant kinetic drug exposures. In this work, we assessed the impact of Agr function on daptomycin activity and lipid metabolism in several genetic backgrounds and in static time-kills and *in vitro* pharmacokinetic/pharmacodynamic (PK/PD) models to better understand the contribution of lipid shedding to daptomycin tolerance.

## Materials and Methods

### Susceptibility Testing and Agr Functionality Testing

The susceptibility to daptomycin was evaluated by broth microdilution in accordance with CLSI guidelines (CLSI, 2017). The Agr functionality was tested on BBL^TM^ Trypticase^TM^ soy agar with 5% sheep blood (TSA II; Becton, Dickinson and Company, Franklin Lakes, NJ, United States) as previously described (Sakoulas et al., 2002). Briefly, a 0.5-McFarland suspension of RN4220 was streaked in a line down the center of the agar plate dividing the plate into two halves, and the test strains were streaked from the edge of the agar plate to the center line of RN4220. Hemolysis was examined after overnight incubation at 37°C.

### Static Time-Kill Assay

Overnight cultures of each strain were inoculated into tryptic soy broth (TSB, Remel Lenexa, KS, United States) supplemented with 50 µg/ml of elemental calcium and 20 µg/ml of daptomycin (Merck, Kenilworth, NJ, United States) to ∼10^8^ CFU/ml in 50 ml conical tubes, incubated at 37°C with shaking. Samples were taken at 0, 2, 4, 6, 8, and 24 h, serially diluted and spiral plated on tryptic soy agar (TSA; Becton, Dickinson and Company, Franklin Lakes, NJ, United States) plates to evaluate the bacterial growth over time with exposure to daptomycin. Experiments were performed under lower aeration (30 ml of culture in 50 ml tube shaken at 85 rpm; SH1000 and SH1001, JE2 and JE2 Δ*agr*) and higher aeration (9 ml of culture in 50 ml tube shaken at 180 rpm; SH1000, SH1001, and SH1000-, USA300 LAC and USA300 LAC Δ*agrA*) conditions, the latter of which were more consistent with the methods by (Pader et al., 2016). All experiments were performed in duplicate.

Lipid Profiling of Static Time-kill of SH1000, SH1001, and SH1000- Overnight cultures of SH1000, SH1001, and SH1000- were inoculated into TSB containing 50 µg/ml of elemental calcium to ∼10^8^ CFU/ml in 50 ml conical tubes, with or without exposure to daptomycin (20 µg/ml) and incubated at 37°C and 180 rpm with a total media culture of 9 ml. Each strain was grown in triplicate for 6 h and pelleted by centrifugation, with 5 ml of the supernatant saved for lipid profiling of the broth. The pellets and the broth were dried in a SpeedVac vacuum concentrator (Thermo Fisher Savant, Waltham, MA, United States), the pellets weighed, and both stored at −80°C until analysis. Lipid extraction, hydrophilic interaction liquid chromatography-ion mobility-mass spectrometry (HILIC-IM-MS), and data analysis were performed as previously described (Hines et al., 2017; Hines et al., 2020), using a Waters Synapt G2-Si ion mobility-QTOF mass spectrometer (Waters Corp., Milford, MA, United States) equipped with an electrospray ionization (ESI) source.

### *In Vitro* Pharmacokinetic/Pharmacodynamic Model and Lipid Profiling

A one-compartment glass model was used to test the impact of a simulated daptomycin exposure on the survival and lipid shedding profile of SH1000 and SH1000-, as previously described (Hall Snyder et al., 2016; Werth et al., 2021). The model apparatus was prefilled with cation adjusted Mueller-Hinton-II broth (MHB; Becton, Dickinson and Company, Franklin Lakes, NJ, United States) supplemented with 50 µg/ml of elemental calcium, and fresh medium was continuously added and removed from the compartment along with the drug *via* a reciprocating syringe pump network (New Era Pump Systems Inc.), set to simulate the average plasma half-life of daptomycin (8 h). The starting inoculum was ∼8 log_10_ CFU/ml. Daptomycin was administered at 0 and 24 h as a bolus injection to achieve the average peak free-drug concentration (C_max_) associated with a 10 mg/kg/day dose (11.3 mg/L) (Benvenuto et al., 2006). All models were performed in duplicate and run continuously for 48 h. All effluent (21.6 ml/h per replicate) was collected from 4 to 5 h and 28–29 h and centrifuged to remove cells. The supernatant was divided into four technical replicates, and subjected to lipid profiling as described above.

## Results

### Not All Agr-Deficiency Slowed Down the Killing of *Staphylococcus aureus* by Daptomycin

**FIGURE 1:**
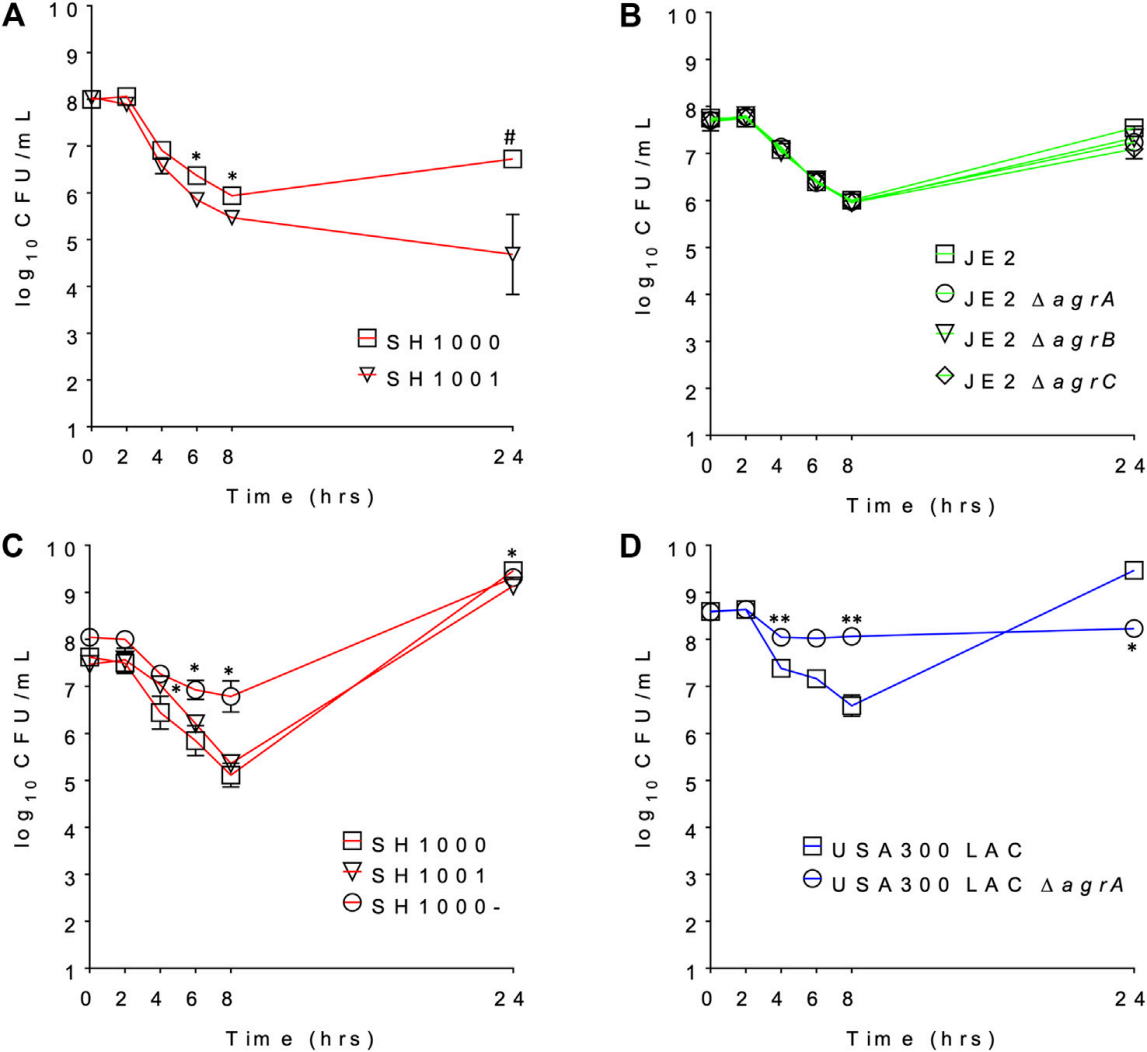
The daptomycin time kill profile of **(A)** SH1000 and SH1001 pair under lower eration; **(B)** JE2 transposon series under low aeration; **(C)** SH1000, SH1001, and SH1000- series under higher eration; and **(D)** USA300 LAC pair under high aeration. ^#^ *p* < 0.001; **0.001 < *p* < 0.01; *0.01 < *p* < 0.05 (based on the percent survival at each timepoint, relative to the wild-type, Student’s *t*-test, two-tailed, equal variance).

We compared the survival of *agr* wild-type and *agr*-defective (either KO or mutant) *S. aureus* under daptomycin exposure using three series of isogenic strain pairs (Table 1) (Boles and Horswill, 2008; Tsompanidou et al., 2011; Fey et al., 2013; Pader et al., 2016). Some of these strains have been well characterized previously (Boles and Horswill, 2008; Tsompanidou et al., 2011; Pader et al., 2016), and we also confirmed the Agr function of SH1001 and the transposon mutants of *agrA*, *agrB*, or *agrC* by examining their hemolytic activity (Supplementary Figure S1). The daptomycin minimum inhibitory concentration (MIC) was 0.25–0.5 µg/ml for all strains (Table 1). Daptomycin time-kill curves for each strain series are illustrated in Figures 1A–D. The wild-type SH1000 survived similarly or better than the *agr* KO strain SH1001, under both high and low aeration for 24 h. SH1000 and SH1001 in Figure 1A were grown under lower aeration than in Figure 1C. The higher aeration allowed both strains to re-grow to a higher and similar CFU/mL at 24 h, although the aeration conditions did not impact the general trend of daptomycin killing of wild-type vs. *agr* KO strains. However, Figure 1C also showed that the *agr* mutant SH1000- survived better than SH1000 and SH1001 for 8 h, with 1.7- and 1.4-log_10_ CFU/ml improved survival, respectively, at the 8 h timepoint. In USA300 LAC background the *agr*-KO strain survived better than the wild-type for 8 h, with 1.5-log_10_ CFU/ml improved survival at the 8 h timepoint (Figure 1D). However, JE2 strains demonstrated similar growth among the wild-type and the *agr* KO’s for 24 h (Figure 1B). Overall, SH1000- and USA300 LAC Δ*agrA* displayed a similar trend as observed previously (Pader et al., 2016), but the *agr*-KO SH1001 and transposon *agr*-KO strains did not display improved survival relative to their matching wild-type strains when exposed to 20 µg/ml daptomycin.

**TABLE 1:** The three series of isogenic *S. aureus* strain pairs of *agr* wild-type and *agr*-defective used in this study and their daptomycin minimum inhibitory concentration (MIC).

Parent	MIC (µg/ml)	Mutant	MIC (µg/ml)	Mutation	Source
SH1000	0.25	SH1001	0.25	Full *agr* KO	Dr. Alexander Horswill
		SH1000-	0.5	H174L mutation in AgrA	Dr. Andrew Edwards
USA300 LAC	0.5	USA300 LAC Δ*agrA*	0.5	Full *agrA* KO	
JE2	0.25	JE2 Δ*agrA*	0.25	Transposon KOs	Nebraska transposon mutant library
		JE2 Δ*agrB*	0.25		
		JE2 Δ*agrC*	0.5		

### Lipid Profiles Released by *Staphylococcus aureus* Upon Daptomycin Exposure Did Not Correlate With Agr Genotypes and Killing Profiles by Daptomycin

**FIGURE 2:**
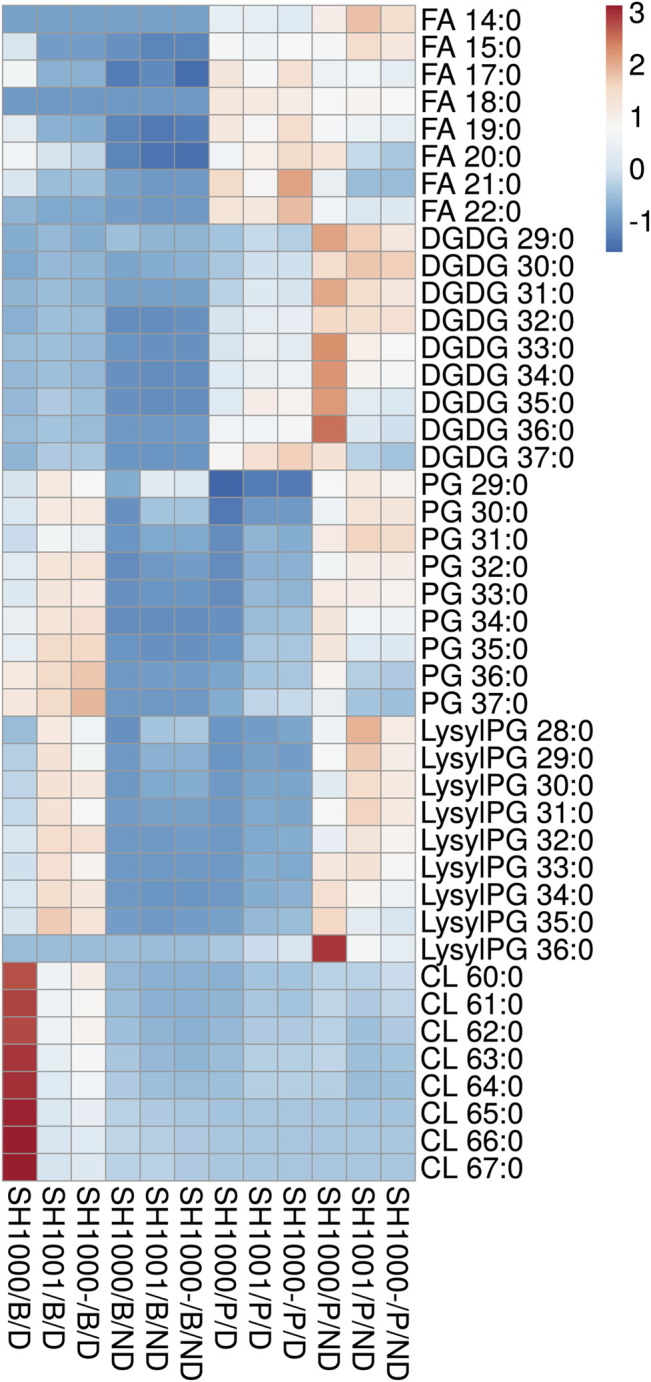
Heatmap of the lipid profile in the broth (B) and the bacterial pellet (P) of the time-kill of SH1000, SH1001 and SH1000-, with (D) or without (ND) daptomycin exposure (row-centered; unit variance scaling applied to rows). Individual lipid species are represented as the number of carbons: the degree of unsaturation in the fatty acid chains. FA, free fatty acid; DGDG, diglucosyl-diacylglycerol; PG, phosphatidylglycerol; LysylPG, lysyl-phosphatidylglycerol; CL, cardiolipin. *N* = 3 per group. See Supplementary Table S2 for *p* values from Student’s *t*-test analysis.

SH1000, SH1001 and SH1000- were grown for 6 h in static time-kills in the presence and absence of 20 µg/ml of daptomycin, and comprehensive lipidomics were carried out on the cell pellets and the broth to profile the membrane lipids and shed lipids. Average dry pellet weights +/− standard deviations of all strains are shown in Supplementary Table S1. The relative abundance of all lipid species including free fatty acids (FAs), diglucosyl-diacylglycerol (DGDGs), PGs, lysylPGs, and CLs were measured and normalized to all compounds. A heatmap depicting the relative abundance of each lipid is shown in Figure 2. All three strains released lipids, regardless of daptomycin exposure, but the levels of shed lipids were higher in the presence of daptomycin. Specifically, the levels of shed PGs were higher with daptomycin exposure than without for all three strains. The Agr-defective strains, SH1001 and SH1000-, shed more PGs than the wild-type SH1000 strain under daptomycin exposure (see Supplementary Table S2 for the complete list of *p* values using Student’s *t*-test). The levels of shed lysylPGs followed a similar trend, except the undetected minor species lysylPG 36:0. CLs were also shed more with daptomycin exposure than without. However, the wild-type SH1000 strain released the largest amount of CLs than SH1001 and SH1000-. FAs and DGDGs were shed only slightly more with daptomycin exposure than without for all three strains, suggesting that PGs, lysylPGs, and CLs are the major lipid classes released in response to daptomycin.

Comparing the levels of shed lipids with the levels of membrane lipids in cell pellets, we found that low levels of shed lipids correlated with high level of membrane lipids, and *vice versa*, regardless of daptomycin exposure, especially for FAs, DGDGs, PGs, and lysylPGs. The same trend was observed for shed and membrane CLs when the three strains were grown under daptomycin exposure. However, when the three strains were grown without daptomycin, the levels of both shed and membrane CLs were relatively low, suggesting CLs might be synthesized and shed specifically in response to daptomycin exposure.

Overall, daptomycin exposure induced more lipids released into the broth, with PGs, lysylPGs, and CLs being the major classes, but the *agr* mutants SH1001 and SH1000- showed similar profile of released lipids, suggesting that the released lipids do not account for their differential killing profiles (Figure 1C).

### Killing Profile in a Pharmacokinetic/Pharmacodynamic Model of Daptomycin Exposure Did Not Correlate With Agr Genotypes

**FIGURE 3:**
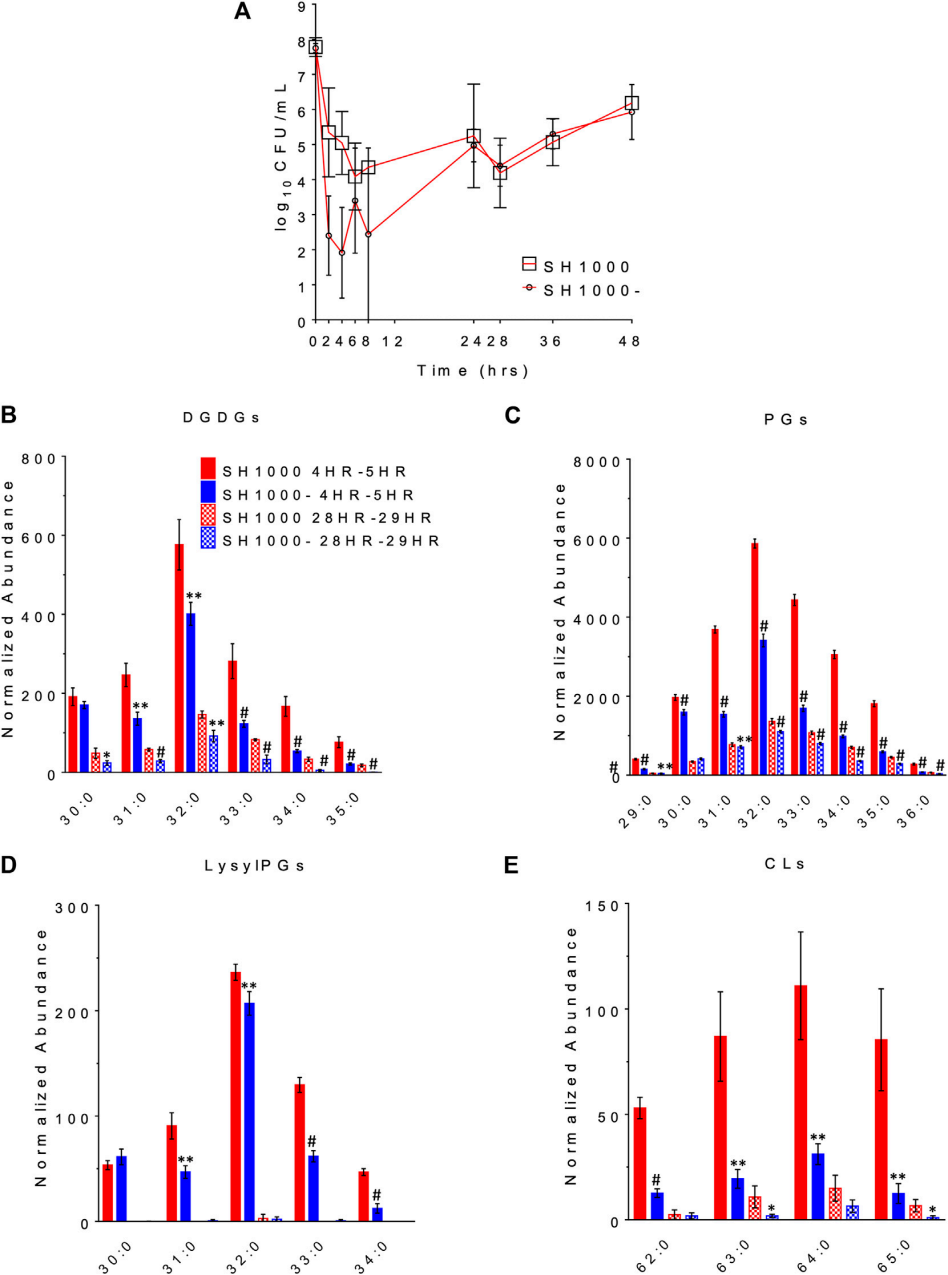
The survival profile **(A)** and lipid profile during 4HR-5HR and 28HR-29HR **(B–E)** of SH1000 and SH1000- in the pharmacokinetics/pharmacodynamics (PK/PD) model of daptomycin exposure. Individual lipid species are represented as the number of carbons: the degree of unsaturation in the fatty acid chains. DGDGs, diglucosyl-diacylglycerols; PGs, phosphatidylglycerols; LysylPGs, lysyl-phosphatidylglycerols; CLs, cardiolipins. ^#^ *p* < 0.001; **0.001 < *p* < 0.01; *0.01 < *p* < 0.05 (Student’s *t*-test, two-tailed, equal variance).

The changes in bacterial densities over time during clinically meaningful kinetic exposures to daptomycin in the PK/PD model are illustrated in Figure 3A. To our surprise, the wild-type SH1000 appear to survive better than the *agr* mutant SH1000- up to 24 h although the difference was not statistically significant (Student’s t-test, two-tailed, equal variance). Furthermore, both grew similarly after the second dose of daptomycin administered at 24 h. These data suggest that lack of Agr function does not provide meaningful advantage to the bacteria under conditions that replicate clinically relevant daptomycin exposures.

The released lipids in the effluent were evaluated at 4–5 h, when the greatest differences in survival were observed, and at 28–29 h, when the wild-type and the *agr* mutant grew back to similar CFU/ml. DGDGs, PGs, lysylPGs and CLs were identified from the lipidomics analysis, as shown in Figures 3B–E (see Supplementary Table S3 for the complete list of *p* values using Student’s *t*-test), among which PGs were the most abundant. Both strains released more lipids at 4–5 h than at 28–29 h, and SH1000 released more than SH1000- at 4–5 h, which seems to correlate with the better survival of the SH1000 strain.

## Discussion

Inactivation of daptomycin by shed lipids of *S. aureus* is an intriguing potential mechanism for daptomycin tolerance. However, after examining the time-kill profiles of *agr* mutant and wild-type strains in three different genetic backgrounds, not all *agr* mutant strains displayed improved survival relative to their isogenic control strains (Figure 1), suggesting that the protection afforded by defective Agr and thus lack of secreted PSMs is not universal. Lipidomic profiling of SH1001 (loss of Agr function due to an *agrA* mutation) (Boles and Horswill, 2008) and SH1000- (full *agr*-KO) (Tsompanidou et al., 2011) showed that both strains released similar lipid profiles to the media (Figure 2) even though their time-kill profiles dramatically differed with only SH1000- displaying better survival than SH1000 (Figure 1C). Furthermore, although SH1001 released more PGs and lysylPGs, but less CLs, than the wild-type SH1000, SH1000 survived better or similarly relative to SH1001 depending on the aeration conditions (Figure 1). These observations suggest that the amount of released lipids does not correlate with the survival of *S. aureus* in the presence of daptomycin.

Comprehensive lipid profiling suggests that phospholipids, including PGs, lysylPGs, and CLs, are preferentially released by *S. aureus* relative to DGDGs and FFAs in response to daptomycin exposure. Furthermore, the lipids released to the media appear to account for relative reductions in the residual membrane lipids in the cell pellets, except CLs. The preferential release of some lipid classes suggests an active releasing process. In particular, daptomycin exposure also upregulates both the synthesis and the release of CLs. This is intriguing as gain-of-function mutation in *cls2*, which encodes cardiolipin synthase, has been associated with daptomycin resistance (Jiang et al., 2019).

In the comprehensive lipidomics analysis of the static time-kills, the average dry pellet weight of the *agr* mutants SH1000- and SH1001 was overall higher than their isogenic wild-type SH1000 with daptomycin exposure (Supplementary Table S1), which is seemingly contradictory to the survival profile (Figure 1C). However, many factors might contribute to the pellet weight, such as the degree of protein synthesis and aggregation of bacterial cells. Additionally, the lipid profile was normalized to all compounds, and hence the differences in pellet weight is unlikely to confound our results.

*S. aureus* and many other bacteria are known to release membrane lipids as extracellular vesicles into their surrounding environment. These released vesicles are composed of lipids, varieties of proteins, polysaccharides, and nucleic acids, and thus may contribute to a variety of biological functions including delivery of intracellular contents for quorum sensing or delivery of virulence factors to host cells (Gurung et al., 2011; Wang et al., 2018). PSMs were found to promote the biogenesis of extracellular vesicles by disrupting cytoplasmic membrane (Wang et al., 2018). *S. aureus* with *psm*α-KO was found to produce significantly less and smaller extracellular vesicles than wild-type. Other factors, such as peptidoglycan cross-linking and autolysis enzymes, also affect the formation of extracellular vesicles. Therefore, it is possible that alternative factors other than PSMs in the extracellular vesicles released by *S. aureus* could contribute to the survival of the bacteria under daptomycin pressure. Elucidation of such factors could shed light on the discrepancy that some *agr* mutant strains resulted in improved survival against daptomycin while others did not.

## Conclusion

In this study, we found that while daptomycin exposure indeed resulted in increased shedding of membrane lipids to the media, the amount and types of released lipids did not correlate with the survival of the bacteria against daptomycin or the genotype of the bacteria. In the cases where there is improved survival, such effect appears to be dependent on experimental conditions, such as aeration, and ultimately are transient effects. Furthermore, the role of the Agr system in counteracting this effect appears to be dependent on the nature of the Agr dysfunction and genetic backgrounds as demonstrated by the variable effects of our isogenic strains with different types of *agr* mutants.

## Data Availability

The original contributions presented in the study are included in the article/Supplementary Material, further inquiries can be directed to the corresponding authors.
