# Supplementary material for: Varied Contribution of Phospholipid Shedding From Membrane to Daptomycin Tolerance in Staphylococcus aureus
Source: Front Mol Biosci. 2021 Jun 11;8:679949. doi: 10.3389/fmolb.2021.679949 (PMC8226217; doi:10.3389/fmolb.2021.679949)
Supplement: Supplementary file 1 [file DataSheet1.PDF]

Supplementary Material for

Varied Contribution of Phospholipid Shedding from Membrane to Daptomycin Tolerance in *Staphylococcus aureus*

Tianwei Shen[1], Kelly M. Hines[1#], Nathaniel K. Ashford[2], Brian J. Werth[2,*], Libin Xu[1,*]

1. Department of Medicinal Chemistry, School of Pharmacy, University of Washington, Seattle, WA, USA

2. Department of Pharmacy, School of Pharmacy, University of Washington, Seattle, WA, USA

[#] current address: Department of Chemistry, University of Georgia, Athens, GA, 30602, USA

* Correspondence:

Brian J. Werth, PharmD, bwerth@uw.edu;

Libin Xu, PhD, libinxu@uw.edu

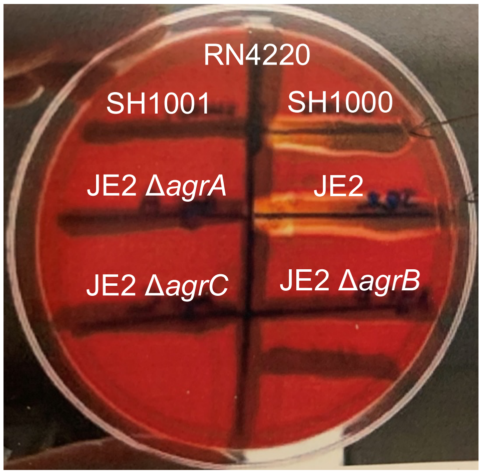

Figure S1. Hemolytic activity of SH1001 and the transposon mutants of *agrA*, *agrB* and *agrC*.

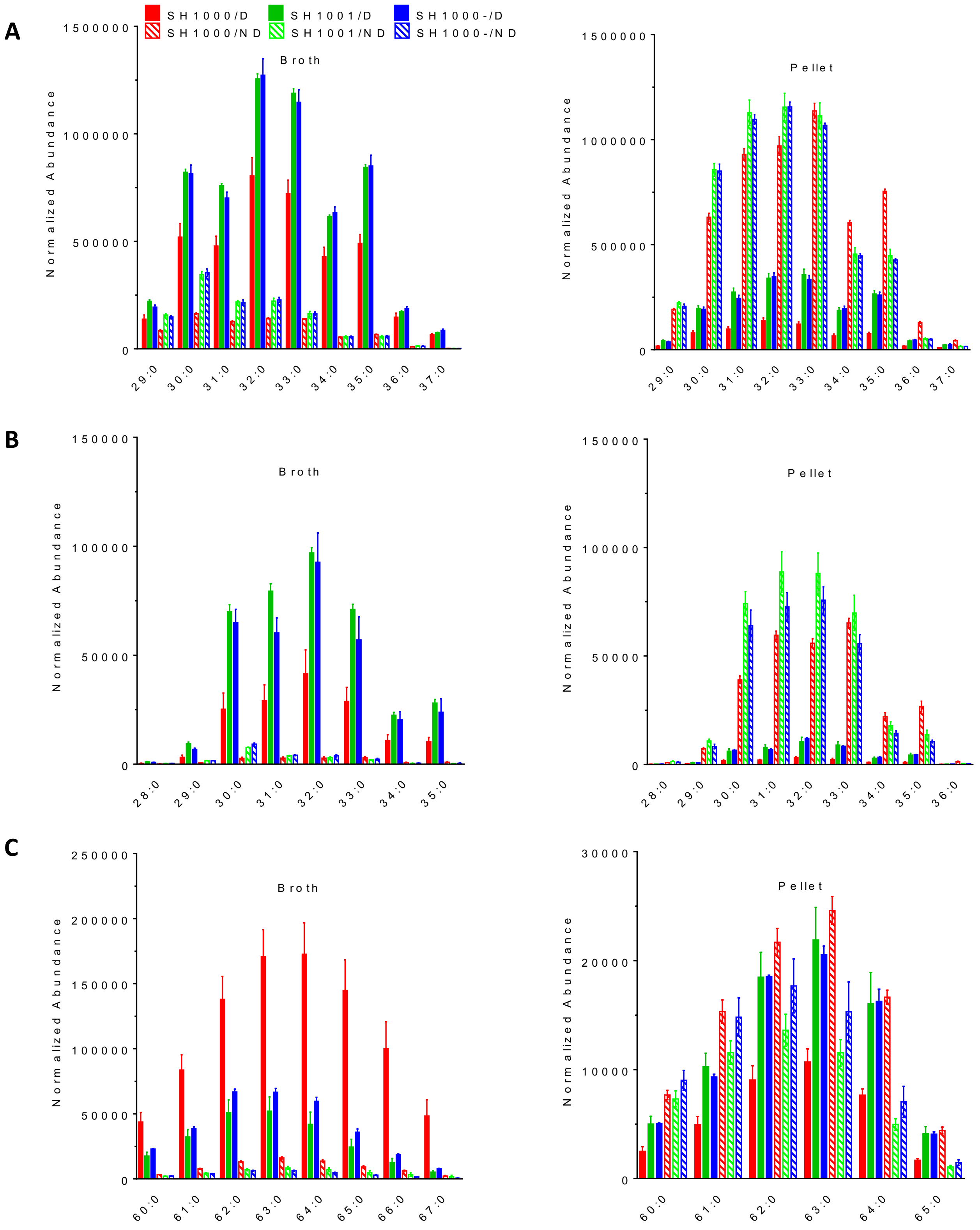

Figure S2. The phophatidylglycerols (**A**), lysyl-phosphatidylglycerols (**B**) and cardiolipins (**C**) profiles in the broth (left) and the bacterial pellet (right) of the time-kill of SH1000, SH1001 and SH1000-, with (D) or without (ND) daptomycin exposure. Individual lipid species are represented as the number of carbons: the degree of unsaturation in the fatty acid chains.

Table S1. Average dry pellet weights +/- standard deviations of all strains for the comprehensive lipidomics analysis of the static time-kills of SH1000, SH1001 and SH1000-. N = 3.

Experimental Group	Average Dry Pellet Weights (mg) ± Standard Deviations (mg)
SH1000, NO DAP	15.1 ± 0.5
SH1001, NO DAP	14.8 ± 0.3
SH1000-, NO DAP	14.6 ± 0.3
SH1000, DAP	3.1 ± 0.4
SH1001, DAP	7.2 ± 0.4
SH1000-, DAP	6.1 ± 0.1

Table S2. Student's *t*-test analysis of the lipids of SH1001 or SH1000- compared to SH1000 in the broth (B) or the bacterial pellet (P) of the time-kill of SH1000, SH1001 and SH1000-, with (D) or without (ND) daptomycin exposure (two-tailed, equal variance). $p<0.001$ is highlighted in red, $0.001<p<0.01$ in blue, and $0.01<p<0.05$ in green. "-" dictates that no corresponding lipid was detected in all three strains under the condition. FA: free fatty acid; DGDG: diglucosyl-diacylglycerol; PG: phosphatidylglycerol; LysylPG: lysyl-phosphatidylglycerol; CL: cardiolipin.

Lipids	*p* Values							
	SH1001/B/D	SH1000-/B/D	SH1001/B/ND	SH1000-/B/ND	SH1001/P/D	SH1000-/P/D	SH1001/P/ND	SH1000-/P/ND
FA 14:0	-	-	-	-	0.927	0.158	0.0166	0.0172
FA 15:0	0.000588	0.00163	0.0539	0.0174	0.325	0.761	0.0181	0.00483
FA 17:0	0.00453	0.00700	0.444	0.283	0.0725	0.647	0.733	0.373
FA 18:0	-	-	-	-	0.863	0.446	0.473	0.882
FA 19:0	0.0171	0.0174	0.105	0.252	0.216	0.319	0.435	0.0515
FA 20:0	0.0337	0.0638	0.000102	0.000430	0.0592	0.0344	0.00150	0.000411
FA 21:0	0.0438	0.0968	0.0137	0.0162	0.0749	0.277	0.00359	0.00390
FA 22:0	0.0153	0.159	0.0365	0.0204	0.649	0.0849	0.0180	0.0323
DGDG 29:0	0.0463	0.327	0.141	0.150	0.0126	0.104	0.000543	0.00707
DGDG 30:0	0.0186	0.00831	0.184	0.0227	0.00560	0.0108	0.00712	0.403
DGDG 31:0	0.0556	0.996	0.409	0.392	0.00285	0.0292	0.00127	0.00444
DGDG 32:0	0.0366	0.0286	0.873	0.150	0.0237	0.0210	0.106	0.280
DGDG 33:0	0.171	0.417	0.00169	0.00217	0.00727	0.0320	0.000145	0.000229
DGDG 34:0	0.0601	0.169	0.000495	0.000388	0.0606	0.0557	1.65E-05	0.000152
DGDG 35:0	0.00825	0.0259	0.000434	0.000401	0.00118	0.00196	1.82E-06	5.16E-06
DGDG 36:0	0.0707	0.512	0.000734	0.000202	0.929	0.542	0.000127	0.000103
DGDG 37:0	0.00522	0.00874	-	-	0.00453	0.000925	0.000537	0.000356
PG 29:0	0.00501	0.0214	9.33E-05	0.000349	0.000992	0.00160	0.00339	0.157
PG 30:0	0.00266	0.00504	5.17E-05	0.000117	0.000453	0.000311	0.000921	0.00112
PG 31:0	0.00104	0.00425	5.18E-05	0.000577	0.000338	0.000324	0.0136	0.00245
PG 32:0	0.00198	0.00448	0.00118	0.000484	0.000347	0.000156	0.0301	0.00636
PG 33:0	0.000552	0.00215	0.0408	0.00685	0.000287	0.000167	0.671	0.0599
PG 34:0	0.00436	0.00554	0.567	0.151	0.000327	0.000182	0.00275	0.000115
PG 35:0	0.000303	0.00140	0.0371	0.00302	0.000117	6.22E-05	0.000171	2.00E-06
PG 36:0	0.148	0.0599	0.0146	0.00793	0.000744	0.000361	1.92E-05	1.90E-05
PG 37:0	0.0999	0.0273	0.0484	0.00847	0.000217	0.000162	4.57E-05	2.06E-05
LysylPG 28:0	0.00268	0.00853	0.000851	0.000369	0.122	0.0427	0.00264	0.177
LysylPG 29:0	0.00162	0.00989	0.000953	0.000768	0.00194	0.000322	0.00417	0.291
LysylPG 30:0	0.00148	0.00438	0.000182	0.000155	0.00461	7.71E-05	0.000984	0.00875
LysylPG 31:0	0.000873	0.0115	0.0578	0.0327	0.00380	0.000129	0.0120	0.0541
LysylPG 32:0	0.00218	0.0140	0.500	0.125	0.00585	8.67E-06	0.00910	0.0122
LysylPG 33:0	0.000985	0.0324	0.0682	0.230	0.00396	0.000111	0.480	0.0458
LysylPG 34:0	0.00511	0.0438	0.0242	0.0810	0.00465	0.000117	0.0790	0.00636
LysylPG 35:0	0.000628	0.0423	0.00973	0.0518	0.00370	5.22E-05	0.00387	0.000716
LysylPG 36:0	-	-	-	-	0.00580	8.21E-05	0.00411	0.00218
CL 60:0	0.00960	0.0164	0.000385	0.000128	0.0123	0.00117	0.582	0.129
CL 61:0	0.00501	0.00567	0.000315	5.41E-05	0.00666	0.00158	0.0247	0.744
CL 62:0	0.00363	0.00478	0.000998	0.000136	0.00709	0.000519	0.00415	0.112
CL 63:0	0.00196	0.00208	0.00183	0.000117	0.00789	0.000625	0.000474	0.0121
CL 64:0	0.00202	0.00277	0.00632	0.000405	0.0150	0.000651	0.0000374	0.000973
CL 65:0	0.00218	0.00286	0.0182	0.000484	0.00762	0.000166	0.000150	0.000482
CL 66:0	0.00407	0.00506	0.0613	0.000562	-	-	-	-
CL 67:0	0.00833	0.0103	0.679	0.00302	-	-	-	-

Table S3. Student's *t*-test analysis of the lipids of SH1001 compared to SH1000- in the broth (B) or the bacterial pellet (P) of the time-kill of SH1000, SH1001 and SH1000-, with (D) or without (ND) daptomycin exposure (two-tailed, equal variance). $0.001<p<0.01$ is highlighted in blue, and $0.01<p<0.05$ in green. "-" dictates that no corresponding lipid was detected in all three strains under the condition. FA: free fatty acid; DGDG: diglucosyl-diacylglycerol; PG: phosphatidylglycerol; LysylPG: lysyl-phosphatidylglycerol; CL: cardiolipin.

Lipids	*p* Values			
	B/D	B/ND	P/D	P/ND
FA 14:0	-	-	0.168	0.138
FA 15:0	0.887	0.894	0.130	0.175
FA 17:0	0.780	0.0207	0.0651	0.158
FA 18:0	-	-	0.584	0.243
FA 19:0	0.810	0.290	0.0973	0.329
FA 20:0	0.561	0.0614	0.131	0.203
FA 21:0	0.838	0.441	0.0277	0.931
FA 22:0	0.7976	0.923	0.0411	0.588
DGDG 29:0	0.0599	0.776	0.0510	0.0575
DGDG 30:0	0.232	0.0202	0.729	0.705
DGDG 31:0	0.0280	0.958	0.0234	0.116
DGDG 32:0	0.311	0.0592	0.560	0.849
DGDG 33:0	0.0291	0.122	0.109	0.140
DGDG 34:0	0.116	0.377	0.695	0.143
DGDG 35:0	0.0347	0.207	0.488	0.0434
DGDG 36:0	0.0692	0.374	0.366	0.0108
DGDG 37:0	0.158	-	0.0283	0.0241
PG 29:0	0.0307	0.223	0.141	0.122
PG 30:0	0.815	0.635	0.750	0.890
PG 31:0	0.0481	0.784	0.156	0.529
PG 32:0	0.779	0.686	0.708	0.984
PG 33:0	0.392	0.906	0.380	0.357
PG 34:0	0.454	0.918	0.518	0.731
PG 35:0	0.867	0.670	0.784	0.410
PG 36:0	0.152	0.519	0.220	0.340
PG 37:0	0.0591	0.646	0.139	0.484
LysylPG 28:0	0.0574	0.420	0.0709	0.0673
LysylPG 29:0	0.0107	0.700	0.327	0.0486
LysylPG 30:0	0.376	0.00698	0.605	0.184
LysylPG 31:0	0.0237	0.260	0.373	0.115
LysylPG 32:0	0.682	0.162	0.336	0.196
LysylPG 33:0	0.143	0.329	0.629	0.0941
LysylPG 34:0	0.502	0.606	0.278	0.0892
LysylPG 35:0	0.406	0.410	0.852	0.0861
LysylPG 36:0	-	-	0.0388	0.0362
CL 60:0	0.0638	0.281	0.978	0.107
CL 61:0	0.193	0.117	0.353	0.0918
CL 62:0	0.0873	0.233	0.970	0.116
CL 63:0	0.144	0.0773	0.569	0.151
CL 64:0	0.0637	0.105	0.931	0.127
CL 65:0	0.0675	0.121	0.972	0.124
CL 66:0	0.0643	0.134	-	-
CL 67:0	0.0290	0.128	-	-
